# Performance improvement for a 2D convolutional neural network by using SSC encoding on protein–protein interaction tasks

**DOI:** 10.1186/s12859-021-04111-w

**Published:** 2021-04-12

**Authors:** Yang Wang, Zhanchao Li, Yanfei Zhang, Yingjun Ma, Qixing Huang, Xingyu Chen, Zong Dai, Xiaoyong Zou

**Affiliations:** 1 School of Chemistry, Sun Yat-Sen University, Guangzhou, 510275 People’s Republic of China; 2 School of Chemistry and Chemical Engineering, Guangdong Pharmaceutical University, Guangzhou, 510006 People’s Republic of China; 3 Research Institute of Sun Yat-Sen University in Shenzhen, Shenzhen, 518000 People’s Republic of China

## Abstract

**Background:**

The interactions of proteins are determined by their sequences and affect the regulation of the cell cycle, signal transduction and metabolism, which is of extraordinary significance to modern proteomics research. Despite advances in experimental technology, it is still expensive, laborious, and time-consuming to determine protein–protein interactions (PPIs), and there is a strong demand for effective bioinformatics approaches to identify potential PPIs. Considering the large amount of PPI data, a high-performance processor can be utilized to enhance the capability of the deep learning method and directly predict protein sequences.

**Results:**

We propose the Sequence-Statistics-Content protein sequence encoding format (SSC) based on information extraction from the original sequence for further performance improvement of the convolutional neural network. The original protein sequences are encoded in the three-channel format by introducing statistical information (the second channel) and bigram encoding information (the third channel), which can increase the unique sequence features to enhance the performance of the deep learning model. On predicting protein–protein interaction tasks, the results using the 2D convolutional neural network (2D CNN) with the SSC encoding method are better than those of the 1D CNN with one hot encoding. The independent validation of new interactions from the HIPPIE database (version 2.1 published on July 18, 2017) and the validation of directly predicted results by applying a molecular docking tool indicate the effectiveness of the proposed protein encoding improvement in the CNN model.

**Conclusion:**

The proposed protein sequence encoding method is efficient at improving the capability of the CNN model on protein sequence-related tasks and may also be effective at enhancing the capability of other machine learning or deep learning methods. Prediction accuracy and molecular docking validation showed considerable improvement compared to the existing hot encoding method, indicating that the SSC encoding method may be useful for analyzing protein sequence-related tasks. The source code of the proposed methods is freely available for academic research at https://github.com/wangy496/SSC-format/.

**Supplementary Information:**

The online version contains supplementary material available at 10.1186/s12859-021-04111-w.

## Background

Deep learning methods are widely used in processing protein–protein interactions (PPIs) and other protein sequence-related tasks, such as potential phosphorylation site identification and protein crystallization prediction from protein sequences [1–10]. In these methods, protein sequences are processed into one hot vectors as the main data format (also called one-of-n coding in some studies), which usually sets an n-dimensional vector, assigns “1” at the index corresponding to the amino acid in the protein sequence, and assigns “0”s at all other positions. This data encoding method plays a fundamental role in deep learning tasks and is the premise to ensure that a reasonable model is well trained and can conduct classification tasks. From a model perspective, as the data type and format determine the performance of a deep learning model, a proper data format can maximize the advantages of a specific model structure; and from a practical perspective, a proper data format leads to a decrease in the system overhead and realizes data fusion from various categories of big data [11, 12].

For one protein sequence with length *L*, the one hot encoding method can transform this sequence (size *L* × 1) into a matrix (size *L* × *n*; commonly, *n* = 20 in protein data). This encoding can expand the dispersed values of the original sequence to Euclidean space, which can improve the calculation of features. Thus, deep learning models (especially CNN models) can extract more abstract features from sequences through convolution operations. Although this approach makes the application of deep learning methods on sequence data possible, there are still some improvements in one hot encoding. One hot encoding is commonly processed with 1D CNNs. In general, 2D CNNs are not used for one hot encoding because 2D convolutional kernels can hardly extract valid features in sparse matrices from a one hot matrix. In addition, the one hot format increases the data size from *L* × 1 to *L* × *n*. In protein-related tasks, 95% of the cells in a one hot matrix are zero. For some complex tasks such as the prediction of the phosphorylation sites or DNA binding sites of proteins, the amount of input data of the CNN model is quite large, especially in analyzing the functions of sequences or PPIs. An appropriate improvement in one hot encoding can provide more abundant information on the basis of a more compact data structure. Accordingly, it is necessary to utilize data reduction in one hot encoding to provide a higher quality data format and reduce the amount of calculation.

As the basis for model training, the format of input data affects the performance and processing scale of the model. Some encoding methods have been introduced to enhance the performance of machine learning methods by improving the format of the input data. Similar to the one hot encoding method, these methods use feature vectors or products for Support Vector Machines (SVMs) and other methods [13]. For example, Martin used a product of signatures to encode proteins [14]. Shen encoded protein sequences as a feature vector to represent the frequencies of 3 amino acid-long subsequences [15]. Guo used autocorrelation values of 7 different physicochemical scales to encode feature vectors for protein sequences [16]. Therefore, these encoding methods increase the accuracy of machine learning methods to varying degrees, which further indicates that the addition of more information into input data can improve performance. This also suggests that the machine learning methods may function more efficiently by applying proper data encoding and thus lead to better results. However, it is difficult to flexibly design proper user-defined data formats for deep learning models due to the complexity of data, and there are still some aspects for further improvement on the common data formats of specific models.

An intuitive way is to keep the input at *L* × 1 while using [1, 2, 3, …, 20] to indicate each amino acid in sequence. However, this format is not commonly reported because these 20 types of amino acids are numerically close to each other and indistinguishable to provide effective features. To increase the discrimination of data features, some methods introduce additional information to improve the model performance. Unbiased dipeptide composition data from the original sequence are used to incorporate unbiased evolutionary profiles and discrete feature spaces [17]. Context-based data are used to calculate statistical moments and are combined with position-related statistical moments to predict the phosphorylation sites [18]. In addition to expressing protein features from amino acid sequences, contextual information is also useful to provide regional characteristics by extending from one amino acid to a sequence fragment. Considering that the original purpose of deep learning methods is to build a neural network for interpreting natural data such as images, sounds and texts, the applications of deep learning methods to process sequence-related data need a proper format to best take advantage of the functional layers in deep learning models [19–21].

In this paper, a new data encoding called the sequence-statistics-content (SSC) format method is proposed to extract information from the original sequence data and improve the performance of deep learning models, especially for protein sequence-related tasks. By extending amino acid composition statistics information and bigram encoding information, a single hot matrix is expanded into multiple channel format. The proposed data format is able to provide more features with additional information, thereby increasing the precision and expanding the application areas of deep learning methods in bioinformatics.

## Result

### Performance evaluation

**Fig. 1:**
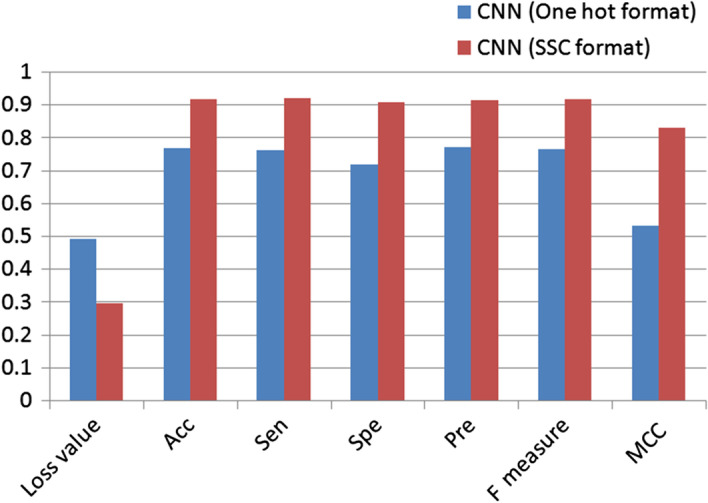
The performance increases from the one-hot format to the SSC format. A CNN model with four convolutional layers was applied to both the SSC format and one hot format. The CNN for the SSC format had a 2D convolution kernel, and the CNN for the one hot format had a 1D convolution kernel

The improvement from the SSC encoding format to the one hot encoding format was obtained by applying a CNN model to the same dataset with different formats. To identify protein–protein interactions, the fivefold cross validation results of the SSC format on the 2D CNN and the one hot encoding on 1D CNN are shown in Fig. 1. Due to adding the statistics and context information from the original sequence, the CNN model increases all the performance indices. The loss value considerably decreases from 0.4916 to 0.2951, the Acc increases from 76.61 to 91.48%, MCC increases from 0.5324 to 0.8295, and the other indexes such as the Sen and F measure also increase by approximately 16%. These results revealed that the performance considerably increased when the SSC format was adopted. This may be attributed to the improvement from integers of "0" and "1" in one hot encoding to floating point values in the SSC format. The SSC format allows more flexible feature extraction in the 2D CNN using 2D convolutional kernels and float values and can provide comprehensive features instead of the discrete features of one hot encoding. The results also indicated that proper format design introducing additional information from protein sequences can enhance the capability of the CNN model to achieve further advancement.

**Fig. 2:**
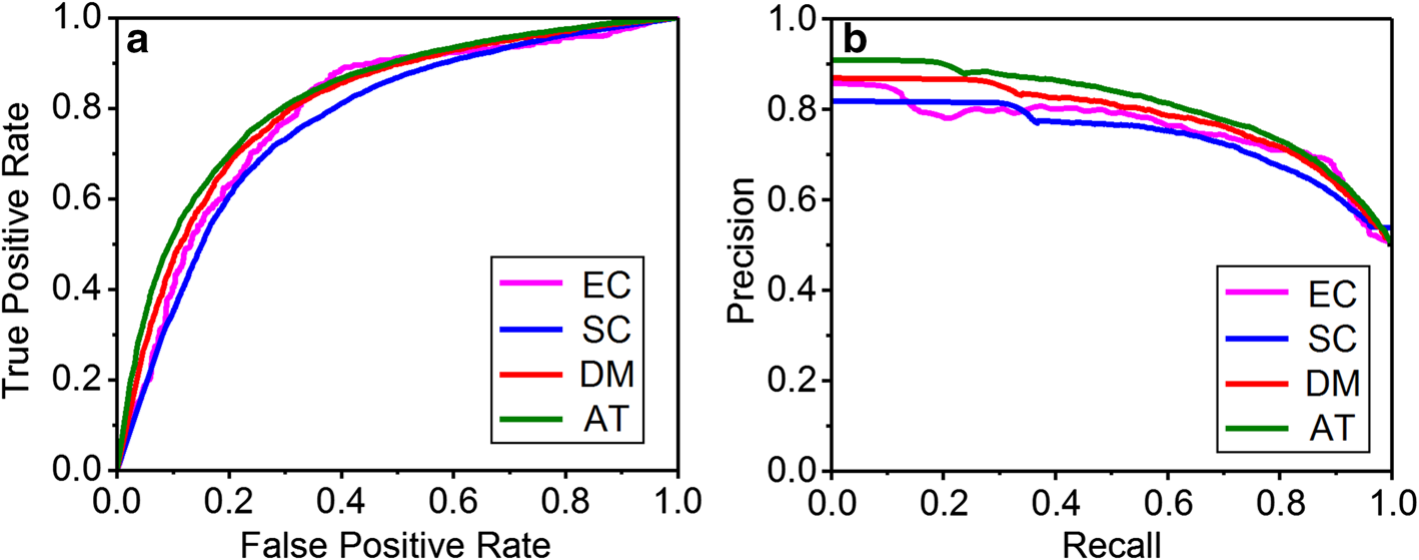
The results of the proposed method on four benchmark datasets. **a** The ROC curves of the predictions on the four benchmark datasets. **b** The PR curves of the predictions on the four benchmark datasets

In order to further evaluate the performance, the CNN model using the SSC encoding format (SSC-CNN) was compared with other methods using four benchmark datasets, including Escherichia coli (EC dataset, 789 proteins with 1752 PPIs), Saccharomyces cerevisiae (SC dataset, 3301 proteins with 21,096 PPIs), Drosophila melanogaster (DM dataset, 1205 proteins with 7718 PPIs) and Arabidopsis thaliana (AT dataset, 1886 proteins with 22,941 PPIs). The results of the SSC-CNN method are listed in Table 1, and the ROC curves and PRC curves are shown in Fig. 2.

**Table 1:** Performance of the SSC-CNN method on four datasets

Dataset	EC	SC	DM	AT
Loss	0.9625	1.0350	0.9743	1.1204
Acc	0.7319	0.7622	0.7429	0.7840
Sen	0.7181	0.7430	0.7263	0.7678
Spe	0.7462	0.8006	0.7602	0.7822
Pre	0.7449	0.7803	0.7583	0.7980
F measure	0.7312	0.7612	0.7420	0.7826
MCC	0.4644	0.5253	0.4866	0.5685

**Fig. 3:**
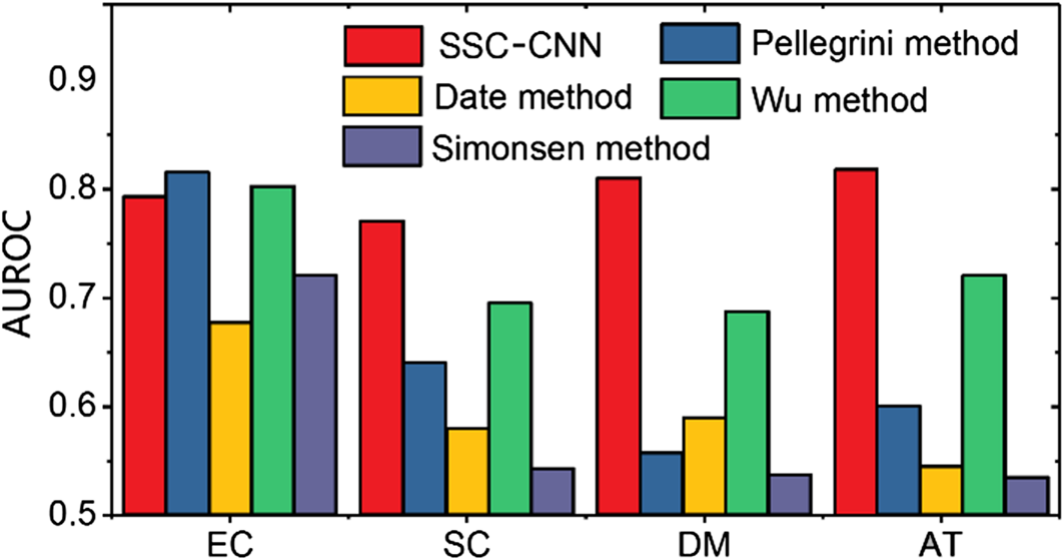
AUROC comparison between the SSC-CNN and the other four methods on benchmark datasets

It can be assumed that the performance of the CNN model depends on the data volume, and the results are shown in Table 1. When small-scale datasets (less than 7800 interactions) are utilized to train the CNN model, the Accs for EC and DM are 73.19% and 74.29%, respectively, and the decrease of approximately 2–5% in comparison with large-scale datasets (more than 21,000 interactions) may be caused by inadequate training for small datasets. The results coincide with the expectation: when a dataset is too small, the performance of the SSC-CNN model is poor and even lower than that of traditional machine learning methods. In addition, with adequate data (HIPPIE dataset, larger than 560,000), the SSC-CNN model can achieve an accurate result (91.48%), indicating that the SSC-CNN method is well suited for analysis with a large dataset scale. As the deep learning method needs sufficient data to train the model, a valid dataset size has a major impact on model training. Besides the quantity, the quality of the dataset is also important EC, SC, DM and AT had unsatisfactory results due to the lower quantity and quality of training data; and the interaction-protein ratios were 2.22, 6.39, 6.40, and 12.16, respectively, compared to HIPPIE (17.07). Next, the SSC-CNN model was compared with the following four methods: (1) the Pellegrini method [22], which employs the Hamming distance between binary phylogenetic profiles to cluster similar profiles; (2) the Date method [23], which uses mutual information between pairs of real-valued profiles as a confidence score; (3) the Wu method [24], which calculates confidence scores for interactions between protein pairs using binary profiles and the hypergeometric distribution; and (4) the Simonsen method [25], which utilizes machine learning based on known PPI networks with phylogenetic profiling. As shown in Fig. 3, except for the EC group, the CNN method on the other three groups can gain a significantly higher AUROC, and it is probable that the performance of the SSC-CNN model on a small-scale dataset (with only 1752 data points) is less than those of traditional methods. The comparison presented here indicated that with the same data, the SSC encoding format can improve the performance of the CNN model, leading to higher prediction capabilities, especially with large datasets.

### Effect of different SSC channel combinations

In order to ascertain the effect of each channel, different channel combinations were investigated separately. The training dataset and test dataset were generated by the following format separately: sequence channel only (channel [S_1_], 60 × 60 × 1), sequence channel and statistics channel (channel [S_1_,S_2_], 60 × 60 × 2), sequence channel and context channel (channel [S_1_,C], 60 × 60 × 2), and all three channels (channel [S_1_,S_2_,C], 60 × 60 × 3). The results of the fivefold cross-validation test based on the various channels are listed in Table 2. Compared with the amino acid channel [S_1_], the extra channels [S_1_,S_2_] and [S_1_,C] improved the model performance; and when both extra channels were used, the [S_1_,S_2_,C] model achieved the best performance for all indexes. These results indicated that the application of an extra channel was effective and can improve the model performance.

**Table 2:** Results of the fivefold cross-validation test based on the various channels

	[S_1_]	[S_1_, S_2_]	[S_1_, C]	[S_1_, S_2_, C]
Loss value	0.3571	0.3453	0.3557	0.2951
Acc	0.8679	0.8699	0.8829	0.9148
Sen	0.8582	0.8799	0.887	0.919
Spe	0.8777	0.8602	0.8789	0.9105
Pre	0.8769	0.8596	0.8785	0.9117
F measure	0.8674	0.8696	0.8827	0.9153
MCC	0.7360	0.7401	0.7659	0.8295

In comparison to channel [S_1_,S_2_], channel [S_1_,C] provided more information about the adjacent amino acids, leading channel [C] to possess higher performance than channel [S_2_]. Furthermore, this adjacent amino acid information resulted in nuanced differences from symmetrical interactions such as protein_a_–protein_b_ and protein_b_–protein_a_. For example, the first twenty amino acids in the sequence of the CNKR1 protein are “MEPVE-TWTPG-KVATW-LRGLD”, which have a centrosymmetric format in channel [S_1_] and channel [S_2_], in both interaction protein_a_–protein_b_ and interaction protein_b_–protein_a_ (e.g., the amino acid order is M–E–P–V–E– in protein_a_–protein_b_ and –E–V–P–E–M in protein_b_–protein_a_, and the percent of each amino acid is symmetric in two interactions); and their features are similar. However, in channel [C], the coding for M–E and E–M are 0.5075 and 0.1750, respectively, which is unsymmetric and thus causes different features. Such interactions in the HIPPIE database were also recorded as different interactions, such as interaction scores of 0.84 for CARF-CD2A1 but 0.82 for CD2A1-CARF. Consequently, this can enhance the prediction accuracy if more asymmetric features are extracted in such cases.

### Effect of the SSC format on the availability of kernels

In the field of image processing, kernels are used to extract and learn image features, and 32 or 64 kernels are usually set in convolutional layers. The SSC format data differ from natural images in adjacent pixels: in natural images, pixels are composed of uninterrupted lines; while in the SSC format, the image pixels are distributed independently but regularly. The results indicated that the application of extra channels was effective due to the more detailed features from the SSC format than sequence data; therefore, kernel scales were ascertained to be the best to make the best use of the extra channels in the SSC format. In some natural image processing, 32 and 64 kernels are sufficient to ensure an accuracy over 85%. Due to the rich amount of data, a large number of kernels are added to process SSC data. In 4 convolutional layers, different kernel scales are tested as followed groups, and the kernel combinations in layer 1 to layer 4 are the following: (1) 32, 32, 64, and 64, respectively; (2) 64, 64, 128, and 128, respectively; (3) 128, 128, 256, and 256, respectively; and (4) 256, 256, 512, and 512, respectively. Uniform initialization and the Adam optimizer were used to train the model, and the results of the fivefold cross-validation test based on the different kernel scales are listed in Table 3. As the number of kernels increased, the Acc, Sen, Spe, Pre, F measure, and MCC indexes were improved considerably, which may be caused by more detailed feature extraction with the addition of more kernels. However, the loss index of the 128/256 kernel combination is similar to that of 64/128 kernels and less than those of 32/64 and 256/512 kernels. Consequently, there is no positive association between the loss value and the number of kernels.

**Table 3:** Results of the fivefold cross-validation test based on the different kernel scales

Index	Kernels			
	32/64	64/128	128/256	256/512
Loss	0.3845	0.2922	0.2951	0.3424
Acc	0.8343	0.9062	0.9148	0.9191
Sen	0.8165	0.9153	0.919	0.9205
Spe	0.8522	0.8969	0.9105	0.9178
Pre	0.8474	0.9013	0.9117	0.9184
F measure	0.8317	0.9083	0.9153	0.9195
MCC	0.6691	0.8125	0.8295	0.8383

The proposed SSC format based on three channels can further characterize features with additional information from the original sequence and thereby improve the availability of more kernels, thus achieving a better result. The cost of more kernels was higher time consumption. In the 32/64 kernel combination, it took only 240 s each epoch for training all 440,000 PPI data points on average and rapidly grew to 1930s when the kernels increased to 256/512. Because the performance under the kernel combination of 128/256 was close to that of 256/512 with only half the time cost (890 s on average), 128/256 kernels were adopted as the common experimental condition.

### Prediction validation

In comparison with the HIPPIE database (version 2.0) in 2016, 53,272 additional interactions involving 11,567 proteins were updated in the database (version 2.1) in 2017. As the effect of the model is closely related to the quality of the dataset, the uneven number of interactions distribution of these proteins exerts a significant impact on the performance of the model. Among 11,567 proteins retrieved from HIPPIE data (version 2.0), the interaction-protein ratio of 8752 proteins was lower than 50, that of 3706 proteins was lower than 10, and the average was approximately 18 (i.e., 287,357 interactions divided by 16,828 proteins). Most of the additional interactions involve one or two proteins with an interaction-protein ratio lower than average, suggesting that the predictive accuracy for these inadequate-information proteins is low. Therefore, a threshold value (i.e., the interaction-protein ratio exceeds a certain value) is utilized to distinguish PPIs in which both proteins have enough interactions with other proteins. In addition, the predictive results of PPIs under different thresholds are listed in Table 4.

**Table 4:** The effect of the threshold on the predictive results

Threshold	Acc	PPIs	PPIs predicted	Proteins
20	0.6930	18,048	12,509	5289
30	0.7915	11,809	9348	3917
40	0.8435	8375	7065	2997
50	0.8805	6193	5453	2372
60	0.9041	4885	4417	1894
70	0.9275	3698	3430	1522
80	0.9439	2908	2745	1253
90	0.9685	2355	2281	1072
100	0.9788	1847	1808	902

As shown in Table 4, the Acc is improved from 0.6930 to 0.9788 as the threshold increases from 20 to 100. When the threshold was set to 20, the Acc was 0.6930, and 12,509 PPIs were correctly predicted among 18,048 PPIs. As discussed before, the predictive results are impacted by the interaction-protein ratio, and the model predictive capability can be steadily enhanced if the average interaction-protein ratio increases. The Acc agreed with expectation and increased to 0.7915 and 0.9788 while the threshold was set to 30 and 100, respectively. The results revealed that the proposed method can gain a higher and reliable predictive result by providing adequate PPIs by setting a suitable threshold. When the proposed method is applied to limited data, to ensure accuracy and reliability, it is necessary to identify other features such as protein active sites and binding sites from the database.

**Fig. 4:**
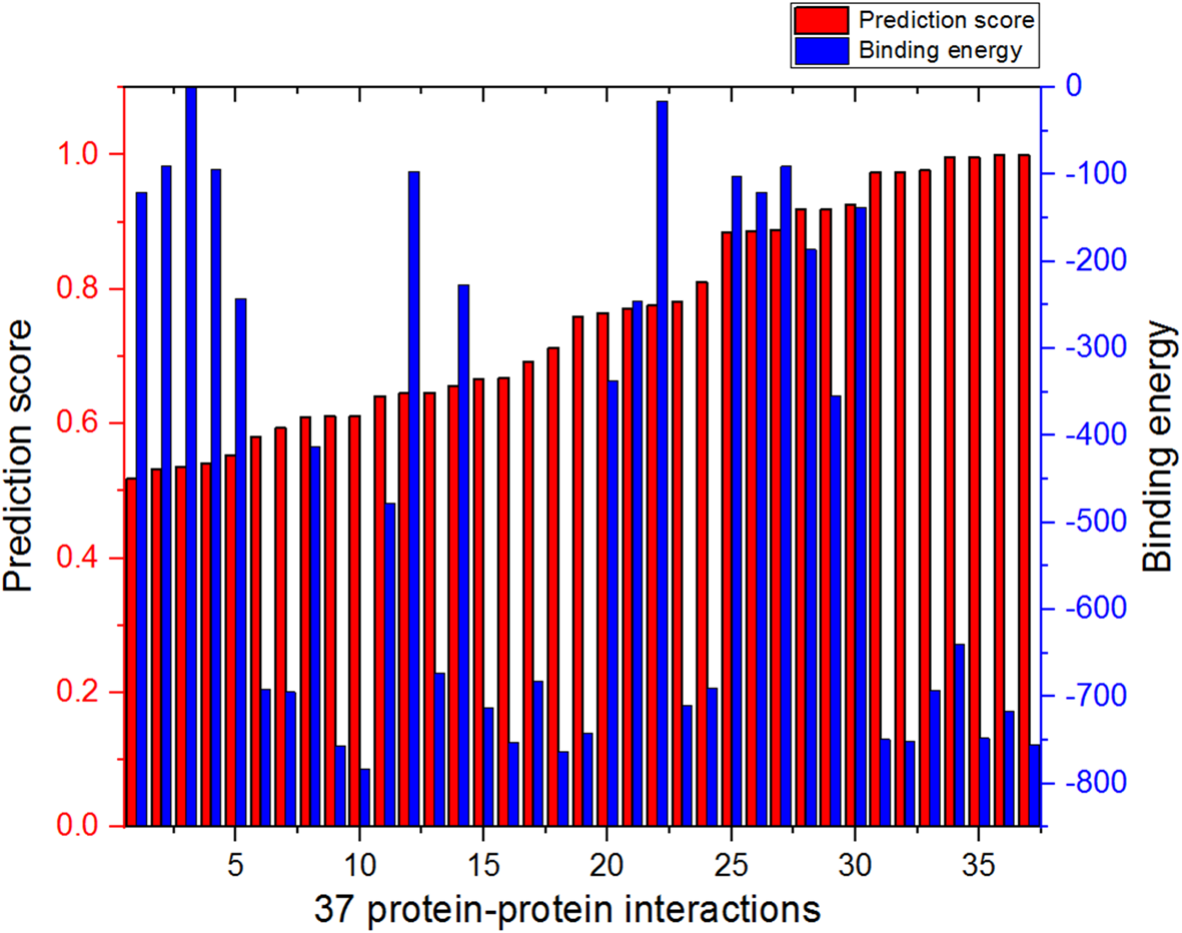
Prediction scores and calculated energy of 37 interactions. The 37 predictions (red columns) are sorted by score, and the energy values (blue columns) reflect the theoretical binding energy of each interaction. The x axis shows the order of 37 PPIs. A higher absolute value of energy suggests more stable binding

To further study and confirm the potential PPIs, we performed an interactive protein docking simulation using the Hex program based on spherical polar Fourier correlations (http://hex.loria.fr/) to calculate the binding energy (i.e., theoretical interaction intensity), and protein structure data (.pdb format) were downloaded from the RCSB Protein Data Bank. Binding energy is the energy released upon the creation of a bound state. A negative binding energy value usually means a possible bound, and a larger absolute value signifies a more stable state of the bound system. Thirty-four proteins in Table S9 were obtained from HIPPIE records with three-dimensional structures, and there were a total of 1156 (34 × 34) combinations for these proteins, where 52 interactions were known and 1104 were unrecorded. The 45 interactions were predicted to be potential interactions because the prediction score was greater than 0.5. Then, Hex was used to calculate the binding energy to validate these 45 predictions. Finally, 37 predictions were calculated to be possible interactions because the binding energy was negative. The results are shown in Fig. 4, and complete details are listed in Table S10. For all possible interactions between any two proteins among these 34 proteins, the results showed that the Acc was near 0.8222, which proved the effectiveness of the proposed method. We noted that not all the higher prediction scores correspond to a larger absolute value of the binding energy, so the proposed method is proper to handle the classification tasks instead of calculating the degree of binding.

## Discussion

We have presented protein sequence encoding methods by extending extra information from the original sequence. More specifically, a multiple channel data format is applied for the 2D CNN model to extract 2D features. The amino acid channel, statistics channel and context channel are obtained and utilized to achieve a higher expression format different from that of traditional one hot encoding. In this way, the classification capability of the deep learning model is accordingly improved by the enrichment of more feature types. The results indicate that this higher abstraction of data characterization is conducive to enhancing deep learning methods. The CNN model using SSC encoding can take advantages of multiple channels (Table 2) and multiple kernels (Table 3) to improve performance. The SSC-CNN can obtain an accuracy of 91.48% on large-scale dataset (contains 521,278 PPIs), and on small-scale the AUC value is also considerable than traditional methods (Fig. 3). The validation results further discuss the application capability on potential PPI, by binding energy from interactive protein docking simulation (Fig. 4).

The improvement of the proposed method can be mainly attributed to these three aspects: first, the traditional protein sequence is extended into the SSC encoding format instead of the one hot encoding format, which enriches the data feature types for the classification of the CNN model; second, the adjacent data layout allows two-dimensional feature extraction with a 2D convolution kernel and many more kernels are fully applied in the calculation than in traditional methods, leading to a classification with more detailed features; and third, extra information from the original sequence increased the distinction between sequences and further improved the efficiency, correctness and reliability of the CNN model.

Compared with traditional encoding methods, the proposed method has some good advantages: more detailed feature extraction leads to a better result; the encoding format can be designed flexibly according to extra information for a specific purpose; and the SSC encoding method for proteins can be easily deployed to other methods or tasks, such as the prediction or analysis of protein folding or protein phosphorylation site prediction. More importantly, the developed method can be flexibly combined with other information, which overcomes the limitation of the data types under deep learning methods. A main limitation of our method is that the expansion of extra channels has demands on a purposeful design. However, the problem can be overcome with experienced researchers or bioinformatics support from the literature.

## Conclusions

In this paper, a PPI prediction method by designing a novel SSC encoding is proposed. The SSC encoding of protein sequence bring considerable improvements for CNN model on PPI task. We believe that the proposed method can provide new insight for applying deep learning methods to bioinformatics, as well as the bridge between the original protein sequence and transformed higher-feature data. It is anticipated that our method may be useful for proteomics studies.

## Methods

### SSC encoding format

The purpose of designing this integrated format is to provide more data features for the CNN model by mining more information from the original sequence or other data sources. Taking the prediction of PPIs as an example, it is necessary to provide sufficient features of sequences to classify whether the two proteins can interact. To introduce more features and reduce the effect from local sequence similarity, two additional data channels are designed from the original sequence, constituting the final three-channel format input data.

The first channel is the amino acid channel, providing sequence information. All 20 types of amino acids from A to Y are encoded as follows:

1 $$Amino\,acid\,channel\left( i \right) = \left[ {\left( {i \times 12} \right) + 20} \right]/255, \quad i = 0, 1,2 \ldots , 19$$

where *i* indicates the *i*th amino acid, and it can directly use 0.05, 0.10, 0.15, …, 1.00 to represent the amino acids from arginine to tyrosine. In order to retain the particularity of sequence features, the amino acid values are designed to be irregular at 0.0784, 0.1255, …, 0.9725, which can also distinguish the values from the third channel.

The second channel is a statistics channel, providing unique features for each protein. The percentage of each amino acid is calculated as:

2 $$Statistics\,channel \left( i \right) = num\left( i \right)/len,\quad i = 0, 1,2 \ldots , 19$$

where *num*(*i*) is the total number of amino acids *i* in the whole sequence, and *len* is the length of this protein sequence. The features from local sequence similarity can be further classified according to different percentages.

The third channel is the context channel. This channel uses bigram encoding to digitize an amino acid pair to provide local context features. The calculation formula is as follows:

3 $$Amino\,acid\,pair \left( {i,j} \right) = \left( {i \times 20 + j} \right)/400,\quad i,j = 0, 1,2 \ldots , 19$$

where *i* and *j* are adjacent amino acids. Adjacent amino acid combinations are utilized to further reduce the local similarity and provide more features for PPI classification. They can both enhance the interaction features and help to classify PPIs. The 20 types of amino acids can provide a total of 400 (20^2^) different values for bigram encoding, which is enough to form features. In addition, there is no need to further use trigrams (20^3^ = 8000) or quadgrams (20^4^ = 160,000) due to the tiny differences of 0.000125 (1/20^3^) and 0.00000625 (1/20^4^), respectively.

**Fig. 5:**
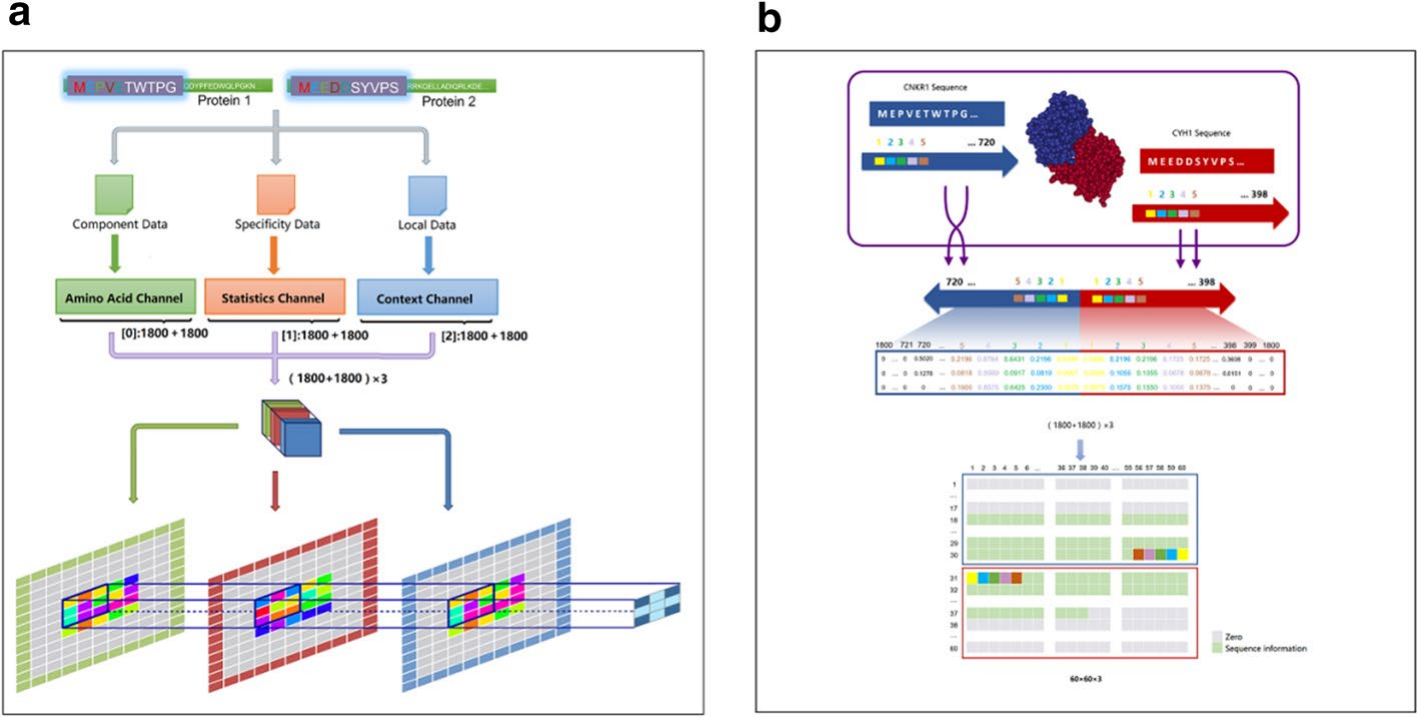
The concept of SSC encoding format. **a** SSC encoding. Three relevant and independent matrices constitute a composite cell. Calculations of features not only possess more combinations from larger numerical ranges but are also promoted by two additional data matrices. **b** Details of 2D protein data conversion. According to the positions of the amino acids in the sequence, each amino acid is assigned a position number. Three channels are filled with each data point, and empty positions are addressed using zero-padding operations. The total length of 3600 × 3 can be reshaped into a 60 × 60 × 3 matrix as the input of the model

After encoding from the protein sequence to the matrix, the sequences are first connected in opposite directions (Fig. 5(B)). Then, two original sequences are extended to a (1800 + 1800) × 3 matrix, and the three rows of the matrix provide sequence information, statistical information, and sequence context information, respectively. The 3600 cells in the matrix are reshaped to 60 × 60, and the three rows turn into three channels. The first channel represents the sequence channel, which provides component data according to the location of each amino acid. The second channel represents a statistics channel, which is another unique sequence note information from the percentage of amino acids and can provide statistical fragment features and discriminate small similar protein fragments. The third channel represents the context channel, which provides local features about sequence information by relating one amino acid to its contiguous amino acid. The calculation details of the three channels are provided in Additional file 1: Section 1, an encoding demonstration of sequence examples is provided in Sections 2.1–2.4, and the final forming of the SSC data is provided in Section 2.5.

### Data collection and preprocessing

The PPI datasets, including 340,629 interaction data points and 16,828 different proteins, were downloaded from HIPPIE [26]. Protein sequence information was obtained from the Uniprot database [27] according to the name of each protein. The 3-D protein structure data were obtained from the RCSB PDB database [28]. Benchmark PPI datasets used to compare the model performance were obtained from the literatures [1, 25].

The PPI prediction task is conducted as the analysis object to test the performance of the proposed data format. In our work, the HIPPIE dataset was utilized as the PPI data. The complete data contain 340,629 interactions and consist of two parts: (1) one part is dataset version 2.0 that contains 287,357 interactions, and these data are utilized to train and test the model (as the training set and test set, respectively); and (2) the other part is an additional new 53,272 interactions from dataset version 2.1, which were considered as independent data to validate model performance.

### Dataset preprocessing

In order to reduce the redundancy of protein sequences and improve the quality of data during the training of the model, the following strategies were performed to handle the sequence data: (1) the proteins were eliminated if the length of one protein sequence was longer than 1800 or shorter than 20 or this sequence information was not available in UniProt. (2) To prevent the overstated calculation caused by sequence redundancy, the CD-HIT suite was used to delete protein sequences with a similarity greater than 60% [29]. Finally, a total of 16,253 proteins and 260,639 PPIs were identified.

A negative sample PPI (i.e., a noninteraction pair between two proteins) is essential for constructing classifiers. The following strategies were used to generate a noninteraction sample, and 260,639 negative samples were finally obtained: (1) Generate random data, which are hypothetical noninteractions, to randomly select from interactions without records in the database. (2) Select data with location information. According to the FASTA information from the UniProt database, if an interaction occurs, two proteins must exist in the same tissues and subcellular locations. Therefore, proteins sharing no tissues and subcellular tissue were collected and considered negative samples, and this part included 70,000 negative samples. (3) Edit data to simulate variation. The 70,000 actual interactions were randomly selected, changing the sequences of these involved proteins. The length of altered sequences ranged from 20 to 80% to simulate various sequence variations. The details are provided in Additional file 1: Section 3. The final 521,279 samples contained 260,639 positive samples, and 260,639 negative samples were randomly sorted. Each sample contained three channels, and each channel was a 60 × 60 matrix. Approximately 80% of the total data (440,000 samples) were divided into a training set, and the remaining 20% (81,278 samples) were used as the test set. The proportion of all positive and negative samples in the training set and test set was 1:1. Considering the comparison with other existing methods, fivefold cross-validation, in which the benchmark dataset is randomly split into 5 subsets, is conducted to evaluate the performance of the model. Every time, four distinct subsets and the remaining subset were utilized to train and test the classifier, respectively.

### CNN model

Among deep learning models such as LSTM [30], the CNN [31], the RNN [32] and the IRNN [33], the CNN has proven its effectiveness with the characteristics of local sense and weight sharing. Local sense is feature extraction in a local area that realizes the abstraction from local sample features to global high-level features. In addition, weight sharing can decrease the amount of calculation and avoid the problem of the curse of dimensionality. In order to extract more features and mine more information from sequence data, a CNN model with four convolutional layers was designed. A flow chart is provided in Additional file 1: Fig. S2, and the details are provided in Section 4. The model optimization details are provided in Additional file 1: Section 5.

**Fig. 6:**
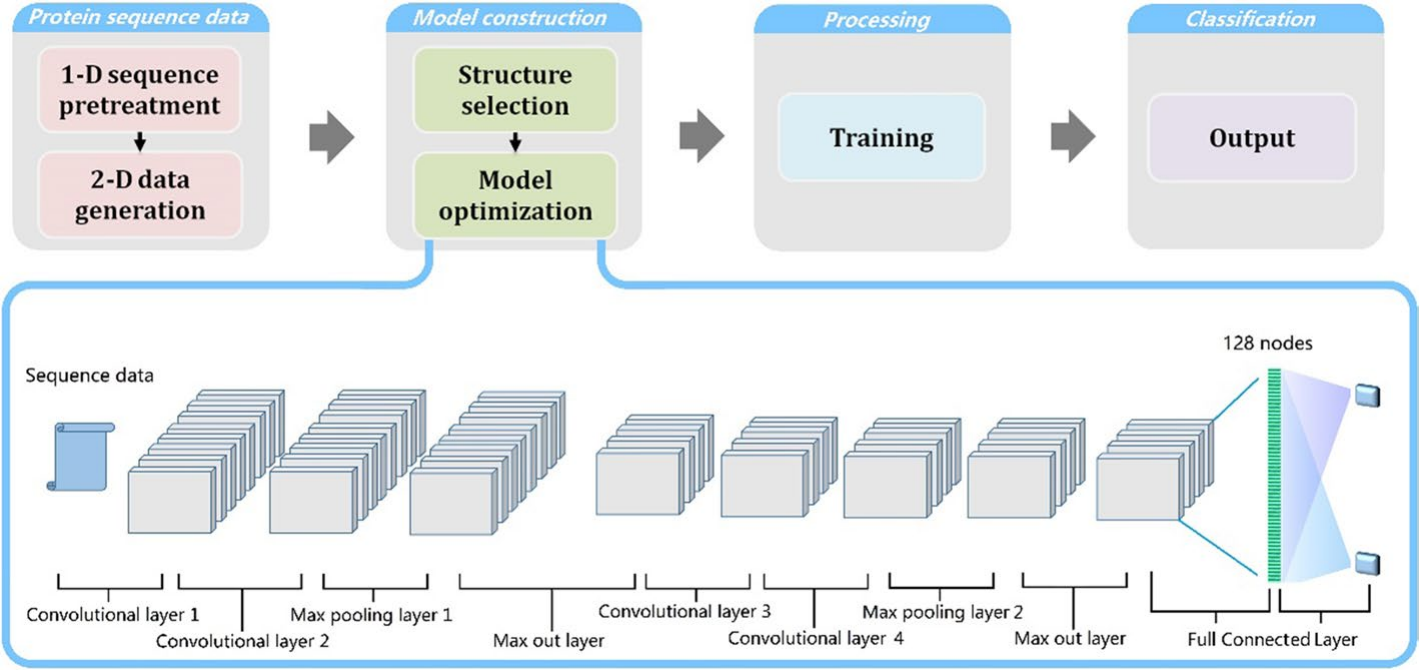
The flowchart and details of the SSC-CNN method

The 521,279 interactions were utilized to generate the training set and the test set. Each interaction dataset contains three channels, and each channel is a 60 × 60 matrix. Therefore, a model designed with 4 convolutional layers convolves from input to output. The structure of the model is shown in Fig. 6. Four convolutional layers are set to extract sufficient features, and one pooling layer is set for every two convolutional layers. The stride is set to 2 in the first two convolutional layers and then increased to 4 to conduct fast shrinking. The dropout is set to 0.25 to prevent overfitting of the model. The 4 convolutional layers with two 2 × 2 pooling layers were constructed in the vertical and horizontal directions. Through a 2-round pooling operation, 10 × 10-dimension features were obtained from 60 × 60 original data to avoid a heavy amount of calculation and overfitting. The data format was set to the last channel, several convolution kernels were set in the 1st and 2nd convolution layers, and kernels were doubly set in the 3rd and 4th convolution layers. Leaky ReLU was utilized as an activation function because it allowed a small gradient when the unit was not active, avoiding gradient deletions caused by some special protein sequences. Leaky ReLU is an improved ReLU function defined as *f*(*x*) = *α* × *x* when *x* < 0 or *f*(*x*) = *x* when *x* ≥ 0, where *α* is a coefficient to adjust the degree of activation of the neuron and to retain some smaller output from the previous layer, which is abandoned in classic activation functions such as SoftMax, ReLU or sigmoid.

### Programming

The program code was written in Python 3.5. The deep learning framework was built with the following packages: Keras (version 2.0.8), NumPy (version 1.13.1) and pandas (version 0.18.1). The main processor is an Intel(R) Xeon(R) Processor E5-2630 v3 and accelerated with an NVIDIA 1080 GPU.

### Performance index

The following indexes were used to evaluate the performance of the proposed method and compare its performance with those of other methods: the accuracy (Acc), sensitivity (Sen), specificity (Spe), precision (Pre), and Matthews correlation coefficient (MCC). Receiver operating characteristic curves (ROCs) and areas under the ROC curves (AUCs) were utilized to estimate the predictive ability and performance [34]. The F measure is the weighted mean of the precision and recall used to balance the error caused by data. Besides, the loss index (Loss) [35] was used to measure the difference between the predicted and real values and can give priority to measure performance while the Acc is similar.

## Supplementary Information

**Additional file 1:** Supplementary information.

## Data Availability

The main codes of this study are freely accessible at the following: https://github.com/wangy496/SSC-format/. The HIPPIE data are freely accessible at the following: http://cbdm-01.zdv.uni-mainz.de/~mschaefer/ hippie/. The Uniprot database is freely accessible at the following: https://www.uniprot.org/. The RCSB PDB database is freely accessible at the following: http://www.rcsb.org/. The Benchmark databases are freely accessible at the following: http://users-birc.au.dk/zxr/phyloprof/. The Hex toolkit is freely accessible at the following: http://hex.loria.fr/. The other data used in this study are included in these published articles.

## Abbreviations

SSC: Sequence-statistics-content
PPI: Protein-protein interactions
CNN: Convolutional neural network
LSTM: Long short-term memory
RNN: Recurrent neural network
IRNN: RNN that is composed of ReLUs and initialized with the identity matrix
Acc: Accuracy
Sen: Sensitivity
Spe: Specificity
Pre: Precision
MCC: Matthew correlation coefficient
AUROC: Area under the receiver operating characteristic curve
ROC: Receiver operating characteristic
PRC: Precision-recall curve
