# Supplementary material for: Performance improvement for a 2D convolutional neural network by using SSC encoding on protein–protein interaction tasks
Source: BMC Bioinformatics. 2021 Apr 12;22:184. doi: 10.1186/s12859-021-04111-w (PMC8042949; doi:10.1186/s12859-021-04111-w)
Supplement: Supplementary file 1 — Additional file 1: Supplementary information. [file 12859_2021_4111_MOESM1_ESM.docx]

**Supplementary Information**

for

Performance improvement for a 2D convolutional neural network by using SSC encoding on protein-protein interaction tasks

Yang Wang ^1^, Zhanchao Li ^2^, Yanfei Zhang^1^, Yingjun Ma^1^, Qixing Huang^2^, Xingyu Chen^2^, Zong Dai ^1,3^, Xiaoyong Zou ^1*,3^

^1^School of Chemistry, Sun Yat-Sen University, Guangzhou, 510275, P.R. China

^2^School of Chemistry and Chemical Engineering, Guangdong Pharmaceutical University, Guangzhou, 510006, P.R. China

^3^Research Institute of Sun Yat-Sen University in Shenzhen, Shenzhen 518000, P.R. China

**1. Calculation results of three channel**

The transformation from protein sequence to binary value is describe as below:

**1.1 Amino acid channel**

Calculation formula for all 20 kinds of amino acid transformation from A to Y is:

Amino acid channel(*i*) = [(*i*×12)+20]/255, *i*=0, 1,2 ..., 19

For example, amino acid of Tyr (Y) and *i*=19, thus the calculation is as below:

Amino acid channel (19) = [(19×12)+20]/255 = 0.9725

The 20 amino acids correspond to 20 values in alphabetical order, and listed in Table S1.

**Table S1.** Amino acid channel for all 20 kinds of amino acid transformation

| No. | Amino acid | Abbreviation | Encoding |
| --- | --- | --- | --- |
| 0 | Arg | A | 0.0784 |
| 1 | Cys | C | 0.1255 |
| 2 | Asp | D | 0.1725 |
| 3 | Glu | E | 0.2196 |
| 4 | Phe | F | 0.2667 |
| 5 | Gly | G | 0.3137 |
| 6 | His | H | 0.3608 |
| 7 | Ile | I | 0.4078 |
| 8 | Lys | K | 0.4549 |
| 9 | Leu | L | 0.5020 |
| 10 | Met | M | 0.5490 |
| 11 | Asn | N | 0.5961 |
| 12 | Pro | P | 0.6431 |
| 13 | Gln | Q | 0.6902 |
| 14 | Arg | R | 0.7373 |
| 15 | Ser | S | 0.7843 |
| 16 | Thr | T | 0.8314 |
| 17 | Val | V | 0.8784 |
| 18 | Trp | W | 0.9255 |
| 19 | Tyr | Y | 0.9725 |

**1.2 Statistics channel**

Statistic formula for all 20 kinds of amino acid from A to Y is:

Statistics channel (*i*) = num(*i*)/*len*, *i*=0, 1,2 ..., 19

Percentage of each amino acid is calculated by this formula, where num(*i*) is total number of one kind of amino acid *i* in the whole sequence and *len* is protein sequence length.

For example, number of amino acid Cys (C) in CNKR1protein sequence is 15, and length of CNKR1 is 720, thus the calculation is as below:

Statistics channel (1) = 15/720 = 0.0208

**1.3 Context channel**

For an amino acid pair, there are totally 20×20=400 possible combinations of two amino acids from A to Y, thus any amino acid pair (*i*, *j*) is calculated as below:

Amino acid pair (*i,j*) = (*i*×20+*j*)/400, *i,j*=0, 1,2 ..., 19

The 20 amino acids correspond to 400 values of amino acid pair (row, column) in alphabetical order, and listed in Table S2. To a protein sequence of length *n*, there are totally *n*-1 amino acid pairs, the corresponding pair value of last amino acid is nonexistent and assigned to the value of zero.

**Table S2.** Context channel for 400 amino acid pairs

|  | A | C | D | E | F | G | H | I | K | L | M | N | P | Q | R | S | T | V | W | Y |
| --- | --- | --- | --- | --- | --- | --- | --- | --- | --- | --- | --- | --- | --- | --- | --- | --- | --- | --- | --- | --- |
| A | 0.0000 | 0.0025 | 0.0050 | 0.0075 | 0.0100 | 0.0125 | 0.0150 | 0.0175 | 0.0200 | 0.0225 | 0.0250 | 0.0275 | 0.0300 | 0.0325 | 0.0350 | 0.0375 | 0.0400 | 0.0425 | 0.0450 | 0.0475 |
| C | 0.0500 | 0.0525 | 0.0550 | 0.0575 | 0.0600 | 0.0625 | 0.0650 | 0.0675 | 0.0700 | 0.0725 | 0.0750 | 0.0775 | 0.0800 | 0.0825 | 0.0850 | 0.0875 | 0.0900 | 0.0925 | 0.0950 | 0.0975 |
| D | 0.1000 | 0.1025 | 0.1050 | 0.1075 | 0.1100 | 0.1125 | 0.1150 | 0.1175 | 0.1200 | 0.1225 | 0.1250 | 0.1275 | 0.1300 | 0.1325 | 0.1350 | 0.1375 | 0.1400 | 0.1425 | 0.1450 | 0.1475 |
| E | 0.1500 | 0.1525 | 0.1550 | 0.1575 | 0.1600 | 0.1625 | 0.1650 | 0.1675 | 0.1700 | 0.1725 | 0.1750 | 0.1775 | 0.1800 | 0.1825 | 0.1850 | 0.1875 | 0.1900 | 0.1925 | 0.1950 | 0.1975 |
| F | 0.2000 | 0.2025 | 0.2050 | 0.2075 | 0.2100 | 0.2125 | 0.2150 | 0.2175 | 0.2200 | 0.2225 | 0.2250 | 0.2275 | 0.2300 | 0.2325 | 0.2350 | 0.2375 | 0.2400 | 0.2425 | 0.2450 | 0.2475 |
| G | 0.2500 | 0.2525 | 0.2550 | 0.2575 | 0.2600 | 0.2625 | 0.2650 | 0.2675 | 0.2700 | 0.2725 | 0.2750 | 0.2775 | 0.2800 | 0.2825 | 0.2850 | 0.2875 | 0.2900 | 0.2925 | 0.2950 | 0.2975 |
| H | 0.3000 | 0.3025 | 0.3050 | 0.3075 | 0.3100 | 0.3125 | 0.3150 | 0.3175 | 0.3200 | 0.3225 | 0.3250 | 0.3275 | 0.3300 | 0.3325 | 0.3350 | 0.3375 | 0.3400 | 0.3425 | 0.3450 | 0.3475 |
| I | 0.3500 | 0.3525 | 0.3550 | 0.3575 | 0.3600 | 0.3625 | 0.3650 | 0.3675 | 0.3700 | 0.3725 | 0.3750 | 0.3775 | 0.3800 | 0.3825 | 0.3850 | 0.3875 | 0.3900 | 0.3925 | 0.3950 | 0.3975 |
| K | 0.4000 | 0.4025 | 0.4050 | 0.4075 | 0.4100 | 0.4125 | 0.4150 | 0.4175 | 0.4200 | 0.4225 | 0.4250 | 0.4275 | 0.4300 | 0.4325 | 0.4350 | 0.4375 | 0.4400 | 0.4425 | 0.4450 | 0.4475 |
| L | 0.4500 | 0.4525 | 0.4550 | 0.4575 | 0.4600 | 0.4625 | 0.4650 | 0.4675 | 0.4700 | 0.4725 | 0.4750 | 0.4775 | 0.4800 | 0.4825 | 0.4850 | 0.4875 | 0.4900 | 0.4925 | 0.4950 | 0.4975 |
| M | 0.5000 | 0.5025 | 0.5050 | 0.5075 | 0.5100 | 0.5125 | 0.5150 | 0.5175 | 0.5200 | 0.5225 | 0.5250 | 0.5275 | 0.5300 | 0.5325 | 0.5350 | 0.5375 | 0.5400 | 0.5425 | 0.5450 | 0.5475 |
| N | 0.5500 | 0.5525 | 0.5550 | 0.5575 | 0.5600 | 0.5625 | 0.5650 | 0.5675 | 0.5700 | 0.5725 | 0.5750 | 0.5775 | 0.5800 | 0.5825 | 0.5850 | 0.5875 | 0.5900 | 0.5925 | 0.5950 | 0.5975 |
| P | 0.6000 | 0.6025 | 0.6050 | 0.6075 | 0.6100 | 0.6125 | 0.6150 | 0.6175 | 0.6200 | 0.6225 | 0.6250 | 0.6275 | 0.6300 | 0.6325 | 0.6350 | 0.6375 | 0.6400 | 0.6425 | 0.6450 | 0.6475 |
| Q | 0.6500 | 0.6525 | 0.6550 | 0.6575 | 0.6600 | 0.6625 | 0.6650 | 0.6675 | 0.6700 | 0.6725 | 0.6750 | 0.6775 | 0.6800 | 0.6825 | 0.6850 | 0.6875 | 0.6900 | 0.6925 | 0.6950 | 0.6975 |
| R | 0.7000 | 0.7025 | 0.7050 | 0.7075 | 0.7100 | 0.7125 | 0.7150 | 0.7175 | 0.7200 | 0.7225 | 0.7250 | 0.7275 | 0.7300 | 0.7325 | 0.7350 | 0.7375 | 0.7400 | 0.7425 | 0.7450 | 0.7475 |
| S | 0.7500 | 0.7525 | 0.7550 | 0.7575 | 0.7600 | 0.7625 | 0.7650 | 0.7675 | 0.7700 | 0.7725 | 0.7750 | 0.7775 | 0.7800 | 0.7825 | 0.7850 | 0.7875 | 0.7900 | 0.7925 | 0.7950 | 0.7975 |
| T | 0.8000 | 0.8025 | 0.8050 | 0.8075 | 0.8100 | 0.8125 | 0.8150 | 0.8175 | 0.8200 | 0.8225 | 0.8250 | 0.8275 | 0.8300 | 0.8325 | 0.8350 | 0.8375 | 0.8400 | 0.8425 | 0.8450 | 0.8475 |
| V | 0.8500 | 0.8525 | 0.8550 | 0.8575 | 0.8600 | 0.8625 | 0.8650 | 0.8675 | 0.8700 | 0.8725 | 0.8750 | 0.8775 | 0.8800 | 0.8825 | 0.8850 | 0.8875 | 0.8900 | 0.8925 | 0.8950 | 0.8975 |
| W | 0.9000 | 0.9025 | 0.9050 | 0.9075 | 0.9100 | 0.9125 | 0.9150 | 0.9175 | 0.9200 | 0.9225 | 0.9250 | 0.9275 | 0.9300 | 0.9325 | 0.9350 | 0.9375 | 0.9400 | 0.9425 | 0.9450 | 0.9475 |
| Y | 0.9500 | 0.9525 | 0.9550 | 0.9575 | 0.9600 | 0.9625 | 0.9650 | 0.9675 | 0.9700 | 0.9725 | 0.9750 | 0.9775 | 0.9800 | 0.9825 | 0.9850 | 0.9875 | 0.9900 | 0.9925 | 0.9950 | 0.9975 |

For example, amino acid pair (Met,Glu) in CNKR1protein sequence is referred to (10,3) according to Table S1, thus the calculation is as below:

Amino acid pair (10, 3) = (10×20+3)/400 = 0.5075

A protein sequence is encoded as 1800 format. If protein sequence length is shorter than 1800, the rest part is assigned to zero. After expanding three channels, two protein sequences are encoded as (1800+1800)×3, and finally reshape to 60×60×3.

**2. Demonstration of protein sequence encoding**

The transformation from amoni acid to encoding value involves three aspects: amino acid mapping as described in Table S1, and context channel as described in Table S2. The whole procedure of transformation is listed in Fig. S1. For example, a PPI between protein CNKR1 (length 720) and CYH1 (length 398) is transformed and part of calculation(sequence fragment of CNKR1 and CYH1 from 1 to 10) is as below:

**2.1 Transformation of amino acid channel**

The transformation of demonstrated fragment sequence refers to Table S2 and transformation of each amino acid is showed in Table S3:

**Table S3.** Transformation of amino acid channel

| **Protein** | **Corresponding transformation for each amino acid** | | | | | | | | |  |
| --- | --- | --- | --- | --- | --- | --- | --- | --- | --- | --- |
| CNKR1 | M | E | P | V | E | T | W | T | P | G |
| Encoding | 0.5490 | 0.2196 | 0.6431 | 0.8784 | 0.2196 | 0.8314 | 0.9255 | 0.8314 | 0.6431 | 0.3137 |
| CYH1 | M | E | E | D | D | S | Y | V | P | S |
| Encoding | 0.5490 | 0.2196 | 0.2196 | 0.1725 | 0.1725 | 0.7843 | 0.9725 | 0.8784 | 0.6431 | 0.7843 |

**2.2 Transformation of statistics channel**

The transformation of statistics channel is based on amino acid number of each full protein sequences, the statistics numbers are showed in Table S4, and transformation results for sequence fragments are showed in Table S5.

**Table S4.** Statistics of CNKR1 and CYH1 protein

| Amino acid | Number inCNKR1 | Percentage | Number in CYH1 | Percentage |
| --- | --- | --- | --- | --- |
| A | 43 | 0.0597 | 21 | 0.0528 |
| C | 15 | 0.0208 | 8 | 0.0201 |
| D | 39 | 0.0542 | 27 | 0.0678 |
| E | 59 | 0.0819 | 42 | 0.1055 |
| F | 16 | 0.0222 | 19 | 0.0477 |
| G | 48 | 0.0667 | 20 | 0.0503 |
| H | 21 | 0.0292 | 6 | 0.0151 |
| I | 19 | 0.0264 | 26 | 0.0653 |
| K | 25 | 0.0347 | 32 | 0.0804 |
| L | 92 | 0.1278 | 36 | 0.0905 |
| M | 7 | 0.0097 | 9 | 0.0226 |
| N | 13 | 0.0181 | 27 | 0.0678 |
| P | 66 | 0.0917 | 16 | 0.0402 |
| Q | 46 | 0.0639 | 15 | 0.0377 |
| R | 44 | 0.0611 | 27 | 0.0678 |
| S | 68 | 0.0944 | 14 | 0.0352 |
| T | 34 | 0.0472 | 19 | 0.0477 |
| V | 41 | 0.0569 | 17 | 0.0427 |
| W | 14 | 0.0194 | 5 | 0.0126 |
| Y | 10 | 0.0139 | 12 | 0.0302 |

**Table S5.** Transformation of statistics channel

| **Protein** | **Corresponding transformation** | | | | | | | | |  |
| --- | --- | --- | --- | --- | --- | --- | --- | --- | --- | --- |
| CNKR1 | M | E | P | V | E | T | W | T | P | G |
| Encoding | 0.0097 | 0.0819 | 0.0917 | 0.0569 | 0.0819 | 0.0472 | 0.0194 | 0.0472 | 0.0917 | 0.0667 |
| CYH1 | M | E | E | D | D | S | Y | V | P | S |
| Encoding | 0.0226 | 0.1055 | 0.1055 | 0.0678 | 0.0678 | 0.0352 | 0.0302 | 0.0427 | 0.0402 | 0.0352 |

**2.3 Transformation of context channel**

The transformation rule refers to Table S2 and transformation of each amino acid pair is showed in Table S6:

**Table S6.** Transformation of context channel

| **Protein** | **Corresponding transformation** | | | | | | | | |  |
| --- | --- | --- | --- | --- | --- | --- | --- | --- | --- | --- |
| CNKR1 | ME | EP | PV | VE | ET | TW | WT | TP | PG | GK |
| Encoding | 0.5075 | 0.2300 | 0.6425 | 0.8575 | 0.1900 | 0.8250 | 0.9400 | 0.8300 | 0.6125 | 0.2700 |
| CYH1 | ME | EE | ED | DD | DS | SY | YV | VP | PS | SD |
| Encoding | 0. 5075 | 0.1575 | 0.1550 | 0.1050 | 0.1375 | 0.7975 | 0.9925 | 0.8800 | 0.6375 | 0.7550 |

**22.4 Data gathering**

The three channel are gathered after transformation encoding, and the results are listed in Table S7 for CNKR1 protein and Table S8 for CYH1 protein.

**Table S7.** Transformed situation of CNKR1 protein

| Amino acid location | Encoding value |
| --- | --- |
| 1 | [0.5490, 0.0097, 0.5075] |
| 2 | [0.2196, 0.0819, 0.2300] |
| 3 | [0.6431, 0.0917, 0.6425] |
| 4 | [0.8784, 0.0569, 0.8575] |
| 5 | [0.2196, 0.0819, 0.1900] |
| 6 | [0.8314, 0.0472, 0.8250] |
| 7 | [0.9255, 0.0194, 0.9400] |
| 8 | [0.8314, 0.0472, 0.8300] |
| 9 | [0.6431, 0.0917, 0.6125] |
| 10 | [0.3137, 0.0667, 0.2700] |

**Table S8.** Transformed situation of CYH1 protein

| Amino acid location | Encoding value |
| --- | --- |
| 1 | [0.5490, 0.0226, 0. 5075] |
| 2 | [0.2196, 0.1055, 0.1575] |
| 3 | [0.2196, 0.1055, 0.1550] |
| 4 | [0.1725, 0.0678, 0.1050] |
| 5 | [0.1725, 0.0678, 0.1375] |
| 6 | [0.7843, 0.0352, 0.7975] |
| 7 | [0.9725, 0.0302, 0.9925] |
| 8 | [0.8784, 0.0427, 0.8800] |
| 9 | [0.6431, 0.0402, 0.6375] |
| 10 | [0.7843, 0.0352, 0.7550] |

**2.5 The whole procedure of transformation**

Each PPI (size 3600) is composed of two protein sequence (size 1800), the corresponding encoding transformations details from one amino acid to numerical value are showed in Supplementary Information Table S2, and rule of context channel is showed in Supplementary Information Table S3. Amino acids are sequenced in the order, each amino acid located by using the following formula, while protein A lined in reverse order of 1~1800 and protein B from 1801 to 3600:

seq_A_(*i*) = format (1799 - *i*);

seq_B_(*j*) = format (1800 + *j*)

An interaction of two protein sequences is integrated into an image sized 3600×1×3 originally. Taking the sequence of protein A for example, seq_A_(*i*) indicates the position *i* for one amino acid in sequence, the relocation of this amino acid in integrated image is calculated by 1799-*i*, procedure for sequence of protein B is performed similarly with A but the relocation in integrated image is 1800+*j*. Protein A constitutes the left half of this format, arranged in reverse order from 1799 to 0, while protein B constitutes the right half of this format from 1800 to the 3599. If the sequence is shorter than 1800, the rest part is zerofilled to prevent the fuzzy padding operation from boundary.


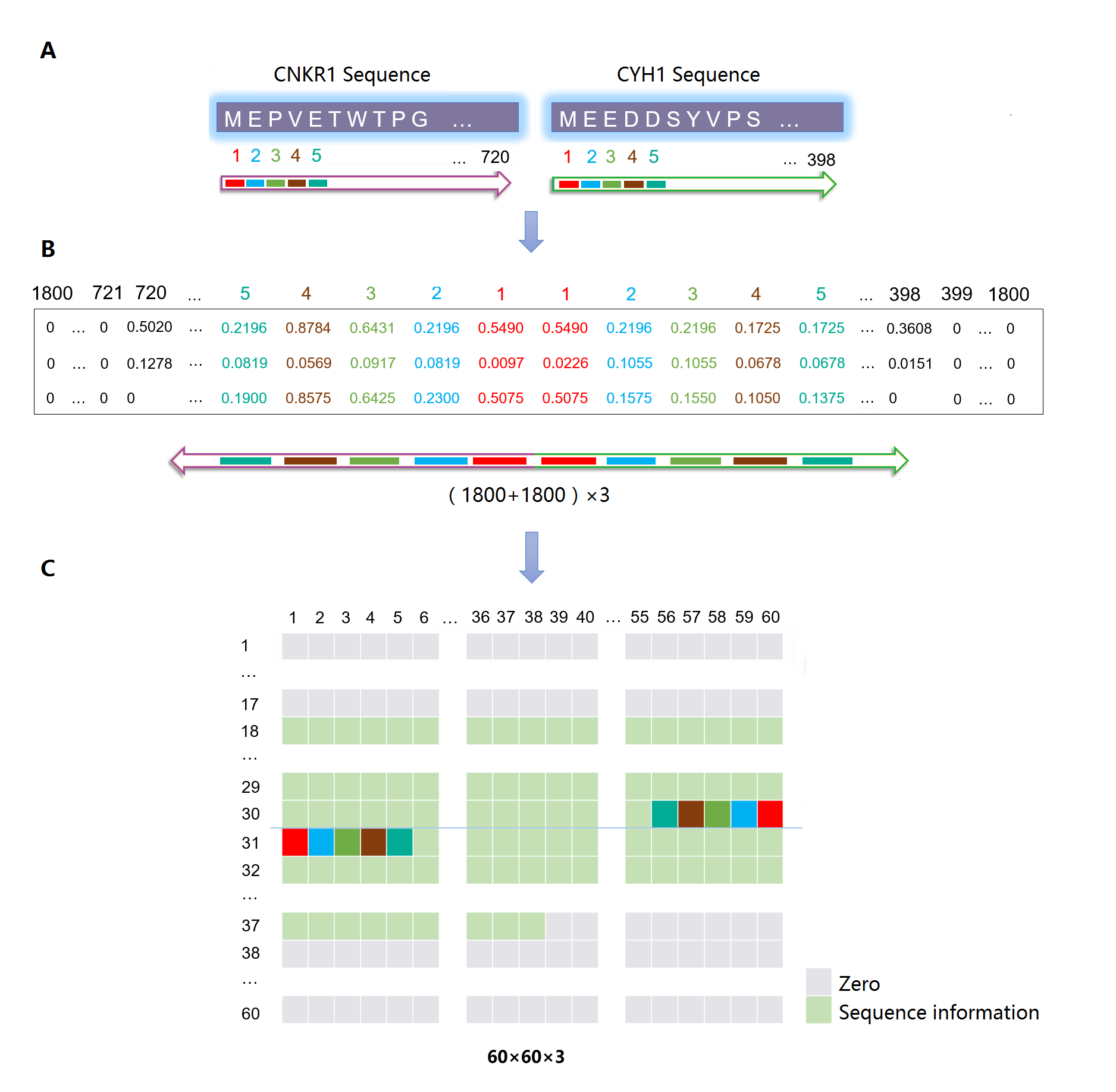


**Fig. S1.** Transforming process from protein sequence to SSC matrix. (A) The two protein sequences are calculated for three channels in the same order, amino acids in CNKR1 and CYH1 sequence are marked in the figure and these two protein sequences are transformed into (1800+1800) format. (B) The two sequences are (1800+1800)×3 matrix by coupling three channels along the reversed direction. (C) Transformation from (1800+1800)×3 matrix to 60×60×3 matrix.

**3. Details of Negative PPI sample generation**

Due to there is no database dedicated to the collection of experimentally verified negative PPI. Therefore, the following strategies were used to generate non-interaction sample: (1) Generating random data, which are hypothetical non-interactions to pick out randomly from interactions without record in database. Since there are over 264 million possible combinations (16253^2^ =264160009) of any two proteins from all 16253 proteins, the interactions matrix is quite sparse and only 0.99‰ elements (260639/264160009) are non-zero. Thus, we randomly selected 120639 interactions that are not contained in the database as negative samples; (2) Selecting data with location information. According to FASTA information from Uniprot database, if an interaction occurs, protein and protein exist mostly in the same tissues or subcellular locations. So, those proteins sharing no tissues and subcellular were collected and considered as negative samples, and this part included 70000 negative samples. (3) Editing data to simulate variation. The 70000 actual interactions were randomly picked out, changing the sequences of these involved proteins. Length of altered sequences ranged from 20% to 80% to simulate various sequence variation.

**4. Detials of PPIs classification structure and CNN model**

The flow chart of PPIs classification and structure of convolutional neural network model is shown in Fig. S2, mainly consisting of four steps:


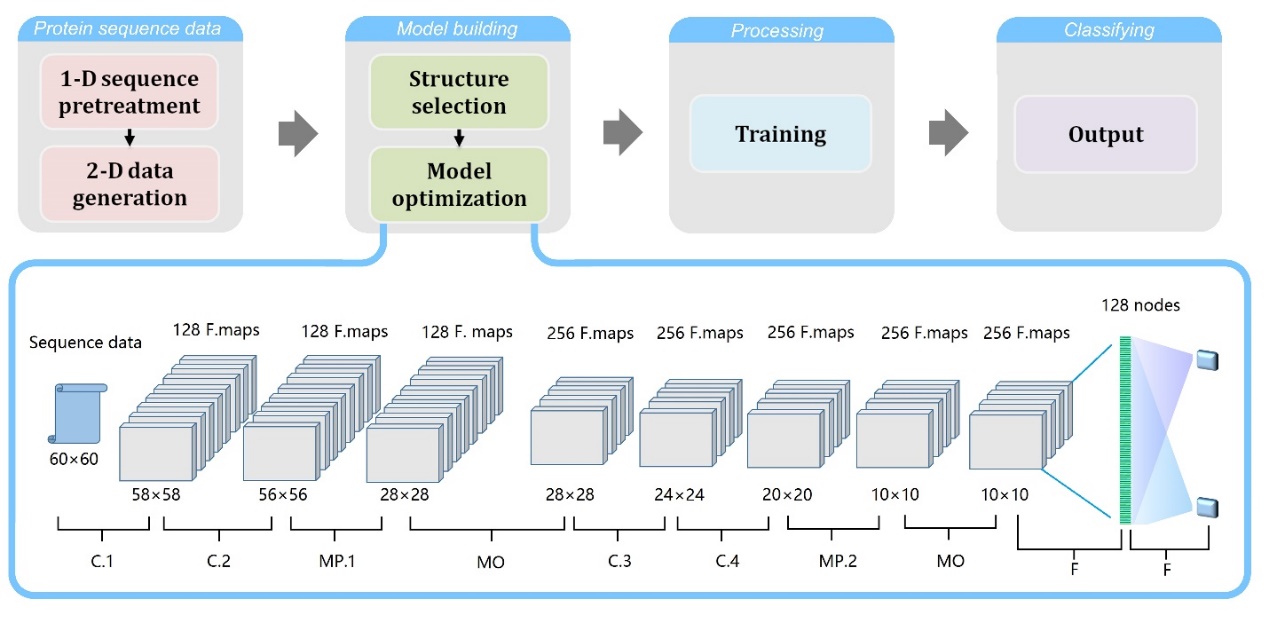


**Fig. S2.** Flow chart of PPIs classification and structure of convolutional neural network model. Input data are preprocessed to 60×60×3 images. After initialization, input are calculated according to C.1 (convolution layer 1) and C.2 (convolution layer 2, image size is cut to 58×58 by 3×3 convolution kernel) layers, and down-conversion is performed through MP.1 (max pooling layer 1, 56×56) layer and MO (max out, 28×28) layer. Then another C.3 (convolution layer 3, 28×28), C.4 (convolution layer 4, 24×24 by 5×5 kernel) layers, MP.2 (max pooling layer 2, 20×20) and MO layer (10×10) are handed with the same way. Finally, the F (fully-connected) layer owns 128 nodes and delivered to a final classification. 128 F. maps and 256 F. maps indicated that there are 128 and 256 kernels in each part to support 128 features and 256 features.

Step 1. Retrieving the information of human PPI from HIPPIE and Uniprot database, respectively. A new SSC data format is designed for PPI data after pretreatments, each valid PPI is decoded into a matrix with three dimensions (i.e. three-channel) including protein sequence information and extending information.

Step 2. Constructing and optimizing deep learning model. Model is built as sequential structure, main layer is composed of two convolutional layers, one pooling layer, one dropout layer, then a flatten layer to flat parameters, and finally a dense layer to output computation result according to model weight. And some parameters are optimized such as initialization, optimizer, and kernels, etc.

Step 3. Training the model with the constructed SSC data to carry out a binary classification for predicting whether there is interaction between two proteins or not.

Step 4. Analyzing the prediction result. The output of model is a prediction score as a tuple [*v_1_*, *v_2_*], which satisfies with *v_1_*+*v_2_*=1 and both *v_1_* and *v_2_* are in the range of [0,1]. Here, *v_1_* represents a non-interaction of the input interactions when categorized to 0, and *v_2_* indicates a true interaction of the input interactions when categorize to 1 while *v_2_*>0.5.

Four convolutional layers in CNN model were set to extract enough features, and one pooling layer for every two convolutional layers. Stride was set to 2 at first two convolutional layers, and then turned to 4 to fast shrink. Dropout was set to 0.25 to prevent overfitting of model. The 4 convolutional layers with 2 pooling layers sized (2×2) were constructed in the vertical and horizontal direction. Through 2 round pooling operation, 10×10 dimension features were obtained from 60×60×3 original data to avoid heavy amount of calculation and over fitting. Data format was set to channel last, and several convolution kernels were set in 1st and 2nd convolution layers, kernels were doubly set in 3rd and 4th. LeakyReLu was utilized as activation function because it allows a small gradient when the unit is not active, avoiding gradient deletions caused by some special protein sequences. It is an improved ReLu function defined as *f*(*x*) =*α* ×*x* when *x* <0 or *f*(*x*) =*x* when *x*≥0, where *α* is a coefficient to adjust activation degree of neuron, and to keep some smaller output from previous layer which are abandoned in classic activation function such as softmax, ReLu or sigmoid.

**5. Model optimization**

A proper initialization is propitious to guarantee the effective updating of weights in model, and decreases the convergence speed; and the optimizer of model directly determines the final performance. To evaluate the effect of different initialization and optimizer on model performance, the 9 initializations and 7 optimizers were tested. These initializations include None (using Keras default setting); Uniform (generated tensors with a normal distribution); Zeros (generated tensors initialized to 0); Ones (generated tensors initialized to 1); Constant (generated tensors initialized to a constant value 0.1); RandomNormal (RN, generated tensors with a normal distribution, the mean of random values was set to 0 and the standard deviation of random values was set to 0.05); RandomUniform (RU, generated tensors with a uniform distribution); TruncatedNormal (TN, generated a truncated normal distribution which is similar to values from a RandomNormal except that values more than two standard deviations from the mean were discarded and re-drawn); Orthogonal (generated a random orthogonal matrix, and multiplicative factor was set to 1); Identity (generated the identity matrix). The results of 5-fold cross-validation test based on the various initializations were listed in Fig. S3(A), details were listed in the Materials and methods section for abbreviations details of performance index. Uniform initialization had the highest Acc and Mcc, and was very close to other best indexes: Sen: 0.8115 to 0.8199 (Identity), Spe: 0.1795 to 0.1734 (RU), Pre: 0.8179 to 0.8239 (RU), and Recall: 0.8115 to 0.8199 (Identity). The F measure index combines measures of Pre and recall, by this index Uniform was higher than RU and Identity. Since the Loss value was similar, Uniform was selected as the initialization of model.

There are several optimizers in the field of image processing and achieve high precision. However, since the unique SSC format is distinguished from the nature images, it is necessary to select a suitable optimizer to deal with this format. Seven optimizers were tested as followed: Rmsprop, SGD, Adam, Adagrad, Adadelta, Adamax, Nadam (Klein *et al.*, 2009; Zeyer *et al.*, 2017). Results of the 5-fold cross-validation test with Uniform initialization based on the various optimizers were listed in Fig. S3 (B). Among these optimizers, Adam got a best index value (Acc, Spe, Pre, F measure and Mcc), Sen was close to Adagrad (0.8165 to 0.8251), and Loss value was similar to each other. Therefore, Adam was selected as the optimizer of model.


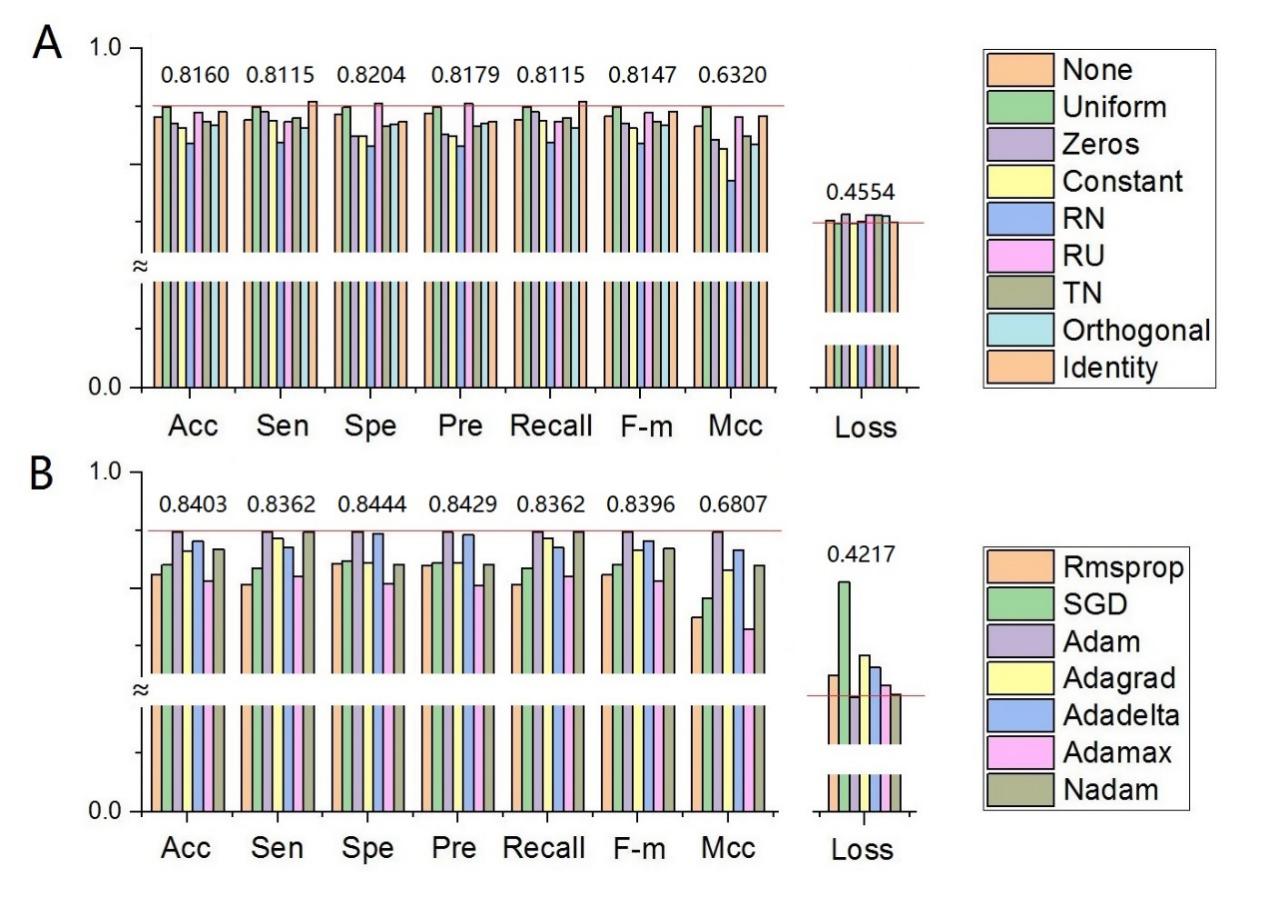


**Fig. S3.** The comparison of initializations and optimizers. (A). Details of performance index among 9 initializations. (B) Details of performance index among 7 optimizers. All the columns in the figure are intercepted from the same histogram(scale from 0 to 1), a base line is set to identify the difference between each column.

**6. Details of validation result**

The 34 proteins with three dimensional structures are obtained from HIPPIE records to validate calculation, the list of 34 proteins are showed in Table S9.

**Table S9.** List of 34 proteins

| No. | Protein |
| --- | --- |
| 1 | CNKR1 |
| 2 | CYH1 |
| 3 | CYH2 |
| 4 | DCR1C |
| 5 | DHE3 |
| 6 | DUS23 |
| 7 | ELL2 |
| 8 | ENAH |
| 9 | GLGB |
| 10 | GLYG |
| 11 | HDAC2 |
| 12 | HNRPC |
| 13 | IL4RA |
| 14 | INP5E |
| 15 | KCRM |
| 16 | LMBL1 |
| 17 | LYN |
| 18 | MTF2 |
| 19 | ODB2 |
| 20 | PAX3 |
| 21 | PCGF6 |
| 22 | PDE6D |
| 23 | PPIP1 |
| 24 | PROF2 |
| 25 | PSA1 |
| 26 | RC3H1 |
| 27 | RPGR |
| 28 | SKP1 |
| 29 | SMG5 |
| 30 | SOSB1 |
| 31 | TBA1A |
| 32 | TFDP1 |
| 33 | TR10A |
| 34 | XRCC4 |

There are 45 predictions in 1156 permutations to be calculated as potential PPIs, the protein involved and calculation are showed in Table S10.

**Table S10.** Details of 45 predictions

| No. | Protein A | Protein B | Prediction score | Binding energy |
| --- | --- | --- | --- | --- |
| 1 | CYH1 | HNRPC | 0.973542 | -750.86 |
| 2 | CYH1 | IL4RA | 0.996077 | -639.39 |
| 3 | CYH1 | PCGF6 | 0.999227 | -755.22 |
| 4 | CYH1 | RPGR | 0.918661 | -186.25 |
| 5 | CYH2 | HNRPC | 0.973542 | -749.33 |
| 6 | CYH2 | IL4RA | 0.996077 | -747.91 |
| 7 | CYH2 | PCGF6 | 0.999227 | -717.35 |
| 8 | CYH2 | RPGR | 0.918661 | -354.05 |
| 9 | DCR1C | IL4RA | 0.646449 | -672.45 |
| 10 | DCR1C | INP5E | 0.64121 | -477.63 |
| 11 | DCR1C | PSA1 | 0.52064 | 482.68 |
| 12 | DHE3 | ENAH | 0.536052 | -0.45 |
| 13 | DHE3 | GLGB | 0.584067 | 28977.63 |
| 14 | DHE3 | HNRPC | 0.888942 | -90.29 |
| 15 | DHE3 | IL4RA | 0.920965 | 211.72 |
| 16 | DHE3 | INP5E | 0.693876 | 0 |
| 17 | DHE3 | MTF2 | 0.598235 | 4659.45 |
| 18 | DHE3 | PCGF6 | 0.645861 | -96.04 |
| 19 | DHE3 | PPIP1 | 0.884211 | -102.17 |
| 20 | DHE3 | RC3H1 | 0.925265 | -137.35 |
| 21 | DHE3 | RPGR | 0.838031 | 7400.66 |
| 22 | DHE3 | SKP1 | 0.541824 | -94.35 |
| 23 | DHE3 | SMG5 | 0.77561 | -15.57 |
| 24 | DHE3 | TR10A | 0.794442 | 7079.3 |
| 25 | HNRPC | CYH1 | 0.611436 | -782.91 |
| 26 | HNRPC | CYH2 | 0.611436 | -755.99 |
| 27 | HNRPC | DHE3 | 0.531471 | -89.79 |
| 28 | HNRPC | ELL2 | 0.552864 | -242.16 |
| 29 | HNRPC | GLGB | 0.764374 | -336.91 |
| 30 | HNRPC | IL4RA | 0.712437 | -763.15 |
| 31 | HNRPC | INP5E | 0.668056 | -752.21 |
| 32 | HNRPC | MTF2 | 0.759822 | -742.58 |
| 33 | HNRPC | PSA1 | 0.770631 | -245.38 |
| 34 | HNRPC | SMG5 | 0.809981 | -690.35 |
| 35 | INP5E | SKP1 | 0.666503 | -713.35 |
| 36 | LYN | IL4RA | 0.580856 | -691.3 |
| 37 | MTF2 | HNRPC | 0.59352 | -694.69 |
| 38 | MTF2 | IL4RA | 0.656866 | -226.51 |
| 39 | MTF2 | RC3H1 | 0.691601 | -682.14 |
| 40 | PDE6D | RC3H1 | 0.609706 | -413.35 |
| 41 | RC3H1 | DHE3 | 0.518577 | -120.3 |
| 42 | RC3H1 | INP5E | 0.885742 | -120.3 |
| 43 | RC3H1 | MTF2 | 0.977172 | -693.59 |
| 44 | SKP1 | IL4RA | 0.781432 | -709.71 |
| 45 | SKP1 | SKP1 | 0.51543 | 35661.64 |
